# Supplementary material for: Pyruvate Dehydrogenase Kinase 1 inhibition mediated oxidative phosphorylation enhancement in cartilage promotes osteoarthritis progression
Source: BMC Musculoskelet Disord. 2023 Jul 20;24:597. doi: 10.1186/s12891-023-06585-6 (PMC10357736; doi:10.1186/s12891-023-06585-6)
Supplement: Supplementary file 1 — Additional file 1. [file 12891_2023_6585_MOESM1_ESM.zip › MF.Gsea.1653627316242/GOMF_2_IRON_2_SULFUR_CLUSTER_BINDING.html]

Details for gene set GOMF\_2\_IRON\_2\_SULFUR\_CLUSTER\_BINDING[GSEA]

|  || Dataset | 55457\_collapsed\_to\_symbols.cls.cls #OA\_versus\_normal.cls.cls #OA\_versus\_normal\_repos |
| Phenotype | cls.cls#OA\_versus\_normal\_repos |
| Upregulated in class | OA |
| GeneSet | GOMF\_2\_IRON\_2\_SULFUR\_CLUSTER\_BINDING |
| Enrichment Score (ES) | 0.6445337 |
| Normalized Enrichment Score (NES) | 1.9135741 |
| Nominal p-value | 0.002247191 |
| FDR q-value | 0.020210978 |
| FWER p-Value | 0.246 |
Table: GSEA Results Summary

  

Fig 1: Enrichment plot: GOMF\_2\_IRON\_2\_SULFUR\_CLUSTER\_BINDING      
 Profile of the Running ES Score & Positions of GeneSet Members on the Rank Ordered List

  

| SYMBOL | TITLE | RANK IN GENE LIST | RANK METRIC SCORE | RUNNING ES | CORE ENRICHMENT || 1 | GLRX5 | glutaredoxin 5 [Source:HGNC Symbol;Acc:HGNC:20134] | 263 | 0.778 | 0.1469 | Yes |
| 2 | FECH | ferrochelatase [Source:HGNC Symbol;Acc:HGNC:3647] | 396 | 0.707 | 0.2888 | Yes |
| 3 | FDX1 | ferredoxin 1 [Source:HGNC Symbol;Acc:HGNC:3638] | 1168 | 0.483 | 0.3328 | Yes |
| 4 | NDUFS1 | NADH:ubiquinone oxidoreductase core subunit S1 [Source:HGNC Symbol;Acc:HGNC:7707] | 1229 | 0.471 | 0.4294 | Yes |
| 5 | ISCU | iron-sulfur cluster assembly enzyme [Source:HGNC Symbol;Acc:HGNC:29882] | 1530 | 0.423 | 0.4971 | Yes |
| 6 | NDUFV2 | NADH:ubiquinone oxidoreductase core subunit V2 [Source:HGNC Symbol;Acc:HGNC:7717] | 1642 | 0.407 | 0.5760 | Yes |
| 7 | SDHB | succinate dehydrogenase complex iron sulfur subunit B [Source:HGNC Symbol;Acc:HGNC:10681] | 1818 | 0.382 | 0.6445 | Yes |
| 8 | BOLA2 | bolA family member 2 [Source:HGNC Symbol;Acc:HGNC:29488] | 4010 | 0.155 | 0.5079 | No |
| 9 | CISD1 | CDGSH iron sulfur domain 1 [Source:HGNC Symbol;Acc:HGNC:30880] | 4104 | 0.146 | 0.5320 | No |
| 10 | ISCA1 | iron-sulfur cluster assembly 1 [Source:HGNC Symbol;Acc:HGNC:28660] | 4116 | 0.144 | 0.5622 | No |
| 11 | CIAPIN1 | cytokine induced apoptosis inhibitor 1 [Source:HGNC Symbol;Acc:HGNC:28050] | 4161 | 0.141 | 0.5892 | No |
| 12 | UQCRFS1 | "ubiquinol-cytochrome c reductase, Rieske iron-sulfur polypeptide 1 [Source:HGNC Symbol;Acc:HGNC:12587]" | 5509 | 0.032 | 0.4915 | No |
| 13 | GLRX2 | glutaredoxin 2 [Source:HGNC Symbol;Acc:HGNC:16065] | 6116 | -0.014 | 0.4474 | No |
| 14 | AOX1 | aldehyde oxidase 1 [Source:HGNC Symbol;Acc:HGNC:553] | 6243 | -0.024 | 0.4427 | No |
| 15 | FXN | frataxin [Source:HGNC Symbol;Acc:HGNC:3951] | 8057 | -0.160 | 0.3364 | No |
| 16 | XDH | xanthine dehydrogenase [Source:HGNC Symbol;Acc:HGNC:12805] | 8366 | -0.183 | 0.3519 | No |
Table: GSEA details [plain text format]

  

Fig 2: GOMF\_2\_IRON\_2\_SULFUR\_CLUSTER\_BINDING      
 Blue-Pink O' Gram in the Space of the Analyzed GeneSet

  

Fig 3: GOMF\_2\_IRON\_2\_SULFUR\_CLUSTER\_BINDING: Random ES distribution      
 Gene set null distribution of ES for **GOMF\_2\_IRON\_2\_SULFUR\_CLUSTER\_BINDING**
